# GOLEM: an interactive graph-based gene-ontology navigation and analysis tool

**DOI:** 10.1186/1471-2105-7-443

**Published:** 2006-10-10

**Authors:** Rachel SG Sealfon, Matthew A Hibbs, Curtis Huttenhower, Chad L Myers, Olga G Troyanskaya

**Affiliations:** 1 Department of Computer Science, Princeton University, 35 Olden Street, Princeton, NJ, USA; 2 Lewis-Sigler Institute for Integrative Genomics, Princeton University, Carl Icahn Labs, Princeton, NJ, USA

## Abstract

**Background:**

The Gene Ontology has become an extremely useful tool for the analysis of genomic data and structuring of biological knowledge. Several excellent software tools for navigating the gene ontology have been developed. However, no existing system provides an interactively expandable graph-based view of the gene ontology hierarchy. Furthermore, most existing tools are web-based or require an Internet connection, will not load local annotations files, and provide either analysis or visualization functionality, but not both.

**Results:**

To address the above limitations, we have developed GOLEM (Gene Ontology Local Exploration Map), a visualization and analysis tool for focused exploration of the gene ontology graph. GOLEM allows the user to dynamically expand and focus the local graph structure of the gene ontology hierarchy in the neighborhood of any chosen term. It also supports rapid analysis of an input list of genes to find enriched gene ontology terms. The GOLEM application permits the user either to utilize local gene ontology and annotations files in the absence of an Internet connection, or to access the most recent ontology and annotation information from the gene ontology webpage. GOLEM supports global and organism-specific searches by gene ontology term name, gene ontology id and gene name.

**Conclusion:**

GOLEM is a useful software tool for biologists interested in visualizing the local directed acyclic graph structure of the gene ontology hierarchy and searching for gene ontology terms enriched in genes of interest. It is freely available both as an application and as an applet at .

## Background

The wealth of publicly available biological information on gene function necessitates the use of sophisticated data structures to organize, explore, and use this information. The Gene Ontology (GO) consortium has developed a unified vocabulary to clarify the relationships of gene products across species and databases [1]. GO comprises three separate ontologies: molecular function (e.g. kinase activity), biological process (e.g. cell cycle), and cellular component (e.g. nucleus). There are a variety of organism-specific gene ontology annotation files, which associate gene products with GO nodes. These include the Saccharomyces Genome Database (SGD) *S. cerevisiae *annotations [2], the FlyBase *D. melanogaster *annotations [3], the Mouse Genome Informatics (MGI) *M. musculus *annotations [4], and the WormBase *C. elegans *annotations [5].

GO is increasingly used to compare gene products across species and to find functional patterns within groups of genes. The gene ontology hierarchy encapsulates functional homology between genes, which may not be evident from other methods of interspecies gene comparison, such as sequence alignment [6]. An important use of GO is the categorization of large-scale gene expression data to identify salient ontological categories in order to facilitate developing biological or mechanistic hypotheses [1]. The large size of the gene ontology hierarchy (currently more than 19,000 terms) and the large number of annotated genes necessitates the use of software tools to mine the information available in the GO database. It is desirable to have software tools that support visualizing the directed acyclic graph (DAG) structure of the gene ontology hierarchy rapidly and dynamically, and allow searching for enriched nodes based on a list of genes of interest.

Several tools to explore the GO hierarchy exist. The official gene ontology browser, AmiGO, allows the user to browse the tree structure of the gene ontology hierarchy, search for GO term and annotated gene name, and filter searches by species, annotations source, or evidence code. Other tools using the information contained in GO include Gene InfoViz, GoFish, FatiGO, TOGO, DynGO, GOTermFinder, and GoMiner [6-12]. A list of available tools to search the gene ontology hierarchy is available on the gene ontology web site [13].

Although the existing tools for GO visualization and analysis are useful in many contexts, to our knowledge no current system provides an interactively expandable view of the graph structure of GO, allowing the user to focus on a local portion of the gene ontology (Table 1). Specifically, many tools show the structure of gene ontology as a tree, in which one term may appear multiple times. The advantage of this approach is that it provides a simplified visualization of the complicated structure of the gene ontology, in which a single term may have multiple parents and many paths back to the root. However, when gene ontology terms are displayed in a tree, connectivity information present in gene ontology is no longer immediately apparent to the user. To allow the user to investigate the structure of GO fully, software tools are needed that display the gene ontology dynamically as an easily searchable, dynamically interactive DAG.

**Table 1:** Comparison of GOLEM with other gene ontology navigation and analysis tools.

	**Availability**	**Finds statistically enriched GO terms**	**Supports gene-based queries**	**Visualization As Tree**	**Visualization As DAG**	**Visualization As Interactively Expandable DAG**	**Accepts Local Annotations Files**	**Requires Internet Connection**
**AmiGO**	Freely available; HTML-based application	No	Yes	Yes	Yes	No	No	Yes
**Gene InfoViz**	Freely available; web-based application	No	Yes	No	Yes	No	No	Yes
**GOFish**	Freely available; Java applet	No	Yes	Yes	No	No	No	Yes
**FatiGO**	Freely available; web-based application	Yes	Yes	No	No	No	No	Yes
**GO Term Finder**	Freely available; yeast-only web-based version, downloadable generic version	Yes	Yes	Yes	Yes	No	No	Yes
**TO-GO**	Freely available; Java application	No	Yes	Yes	No	No	Yes	Yes (at program startup only)
**DynGO**	Freely available; standalone Java package	No	Yes	Yes	No	No	Yes	No
**GenNav**	Freely available; web-based application	No	Yes	No	Yes	No	No	Yes
**GoMiner**	Freely available; Java application	Yes	Yes	Yes	Yes	No	Yes	No
**GOLEM**	Freely available; application version, applet version	Yes	Yes	No	Yes	Yes	Yes	No

Several existing tools, including AmiGO, GeneInfoViz, GoTermFinder, GOMiner, and GenNav, display the graph structure of gene ontology. However, these tools do not support dynamic browsing of the gene ontology hierarchy. Among these tools, only GOTermFinder and GenNav allow the user to change the graph display to focus on a node of interest. However, neither GOTermFinder nor GenNav permit the user to dynamically expand their view of the GO graph.

Furthermore, many currently available tools cannot load local annotation files, so they are useful only for browsing a pre-determined set of annotations files (Table 1). Most tools also do not limit the local focus to terms which contain annotated genes in the chosen annotations file. Displaying nodes without annotated genes makes it more difficult to see the relationships between terms with annotated genes in the organism of interest.

Another limitation is that most existing software tools are web-based or require an Internet connection. Many web-based tools open new windows or reload the current window every time the user wishes to change the display. Simple tasks such as browsing the gene ontology graph often require network traffic over the server. This makes the usability of many tools highly dependent on the speed of the user's Internet connection and the latency of network traffic to the server.

Finally, many tools are focused on providing either visualization or analysis capabilities, but not both. It is desirable to integrate visualization and analysis capabilities in a single software tool, so that the user can browse and search the directed acyclic graph structure of the gene ontology hierarchy, as well as query for terms enriched in a list of genes of interest. To address these considerations, we have developed GOLEM (Gene Ontology Local Exploration Map), a new gene ontology software tool that allows both dynamic visualization of the GO graph and gene enrichment analysis. GOLEM is publicly available as both a stand-alone application and as an applet.

## Implementation

### User interface

GOLEM is implemented in Java, using Java Swing for the graphical user interface. Therefore, GOLEM is platform independent, and can run on any standard machine with JRE 1.5 or higher. The graph visualization is based on the open-source package OpenJGraph [14]. Both applet and stand-alone versions are available.

### Statistical analysis

P-values are calculated using the hypergeometric distribution [9]. If an annotations file contains N genes, a given GO term has M annotated genes, and the user inputs a list of n genes of interest, the probability of seeing k or more genes of interest annotated to a given GO term is computed as:

p−value=∑j=kn(Mj)(N−Mn−j)(Nn)
 MathType@MTEF@5@5@+=feaafiart1ev1aaatCvAUfKttLearuWrP9MDH5MBPbIqV92AaeXatLxBI9gBaebbnrfifHhDYfgasaacH8akY=wiFfYdH8Gipec8Eeeu0xXdbba9frFj0=OqFfea0dXdd9vqai=hGuQ8kuc9pgc9s8qqaq=dirpe0xb9q8qiLsFr0=vr0=vr0dc8meaabaqaciaacaGaaeqabaqabeGadaaakeaacqWGWbaCcqGHsislcqWG2bGDcqWGHbqycqWGSbaBcqWG1bqDcqWGLbqzcqGH9aqpdaaeWbqaamaalaaabaWaaeWaaeaafaqabeGabaaabaGaemyta0eabaGaemOAaOgaaaGaayjkaiaawMcaamaabmaabaqbaeqabiqaaaqaaiabd6eaojabgkHiTiabd2eanbqaaiabd6gaUjabgkHiTiabdQgaQbaaaiaawIcacaGLPaaaaeaadaqadaqaauaabeqaceaaaeaacqWGobGtaeaacqWGUbGBaaaacaGLOaGaayzkaaaaaaWcbaGaemOAaOMaeyypa0Jaem4AaSgabaGaemOBa4ganiabggHiLdaaaa@4F18@

The user can choose to correct for multiple hypothesis testing by applying Bonferroni correction [9], false discovery rate (FDR) correction [15], or no correction. Further, if a user desires to use an alternate method for calculating significance, the provided source code of GOLEM can be altered to use an implementation of a different algorithm (more information available in the instructions on the GOLEM home page)[16].

## Results and discussion

### Overview of GOLEM

GOLEM displays a local view of the DAG structure of the gene ontology hierarchy. Specifically, given a term of interest to the user (either chosen by the user through term search or found significant in enrichment analysis), GOLEM shows a local portion of the gene ontology graph, centered around this term of interest. By displaying the gene ontology hierarchy as a DAG, GOLEM makes all of the information contained in the local structure of the gene ontology hierarchy visually accessible to the user. An alternative to such a locally focused DAG visualization would be a global view with focusing ability. However, the entire GO hierarchy is very difficult to layout, display, and interpret. Furthermore, many researchers use GO with specific terms in mind, and thus a locally focused view is reasonable from the user perspective. As GOLEM permits the user to interactively browse the graph structure of gene ontology, a local view allows focus on regions of interest while maintaining global graph accessibility.

GOLEM allows the user to search gene ontology by GO term name, GO id, and gene name. GOLEM features a search aid window when the user searches by GO term name. This search aid window displays all term names beginning with the letters that the user has entered, facilitating easy browsing of the gene ontology graph.

GOLEM combines dynamic visualization of the DAG structure of the gene ontology hierarchy with the functionality to search for GO nodes enriched with genes of interest. We anticipate that the combination of analysis and visualization provided by GOLEM will be useful to biology researchers.

GOLEM requires network traffic only while reading in GO term or annotations data. Since GOLEM stores GO term and annotation information locally while the program is running, navigating the gene ontology hierarchy is rapid.

Additionally, the GOLEM application provides the user with the option to read gene ontology terms and/or annotations from local files. Therefore, the GOLEM application may be used without an Internet connection. It is possible for the user to input any annotations or ontology file in standard format, including personal ontology or annotations files that have not yet been made publicly available. Alternately, the user may choose to download annotations and ontology files from the gene ontology web page. This ensures that the most recent version of these files is used, an important feature because gene ontology terms are updated frequently.

### Basic functionality

#### Graph visualization

**Figure 1:**
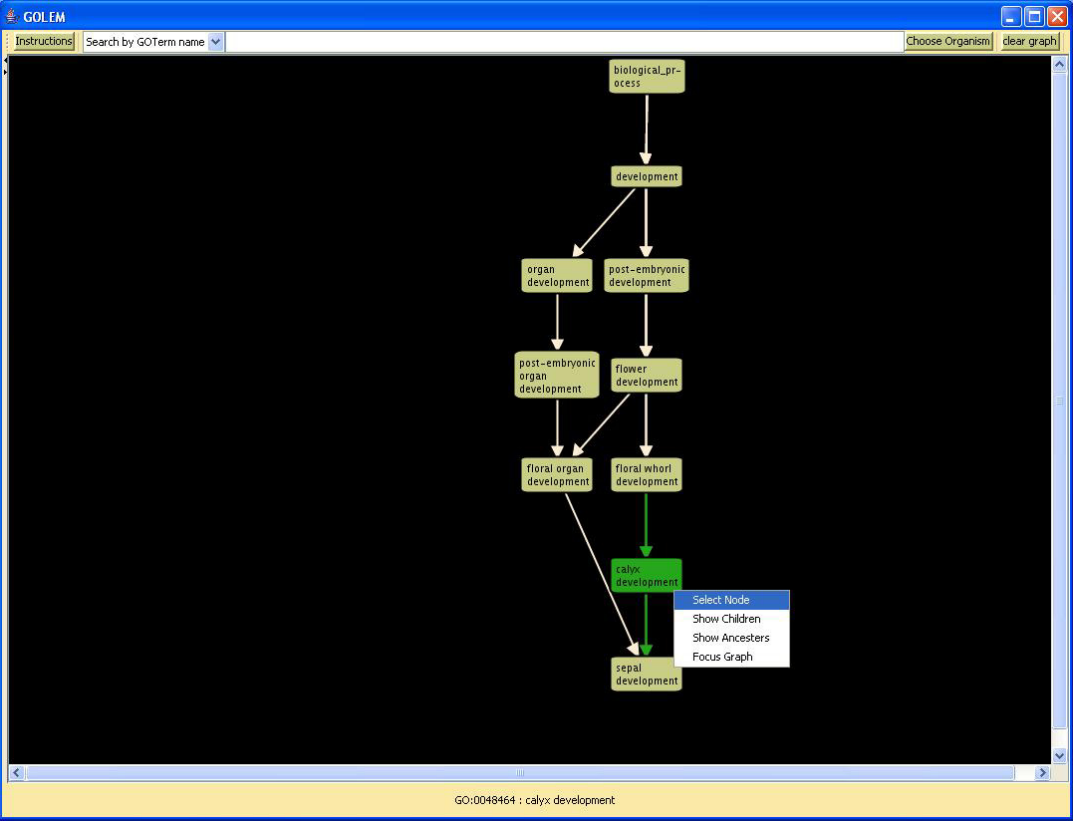
**GOLEM interactively displays the structure of the GO hierarchy in the neighborhood of a selected term**. Searching for the GO term "calyx development" displays the term, all child nodes, and all paths to the root node. The selected term and all incident edges are highlighted. Clicking on a node displays a popup menu that allows the user to dynamically browse the GO graph.

GOLEM allows the user to visualize the directed acyclic graph structure of the gene ontology hierarchy, centered around a selected node (Figure 1). GOLEM focuses on a local portion of the graph and implements a depth-based layout. Specifically, nodes are arranged vertically according to their distance from the root node, and nodes with greater degree are centered.

#### Interactivity

**Figure 2:**
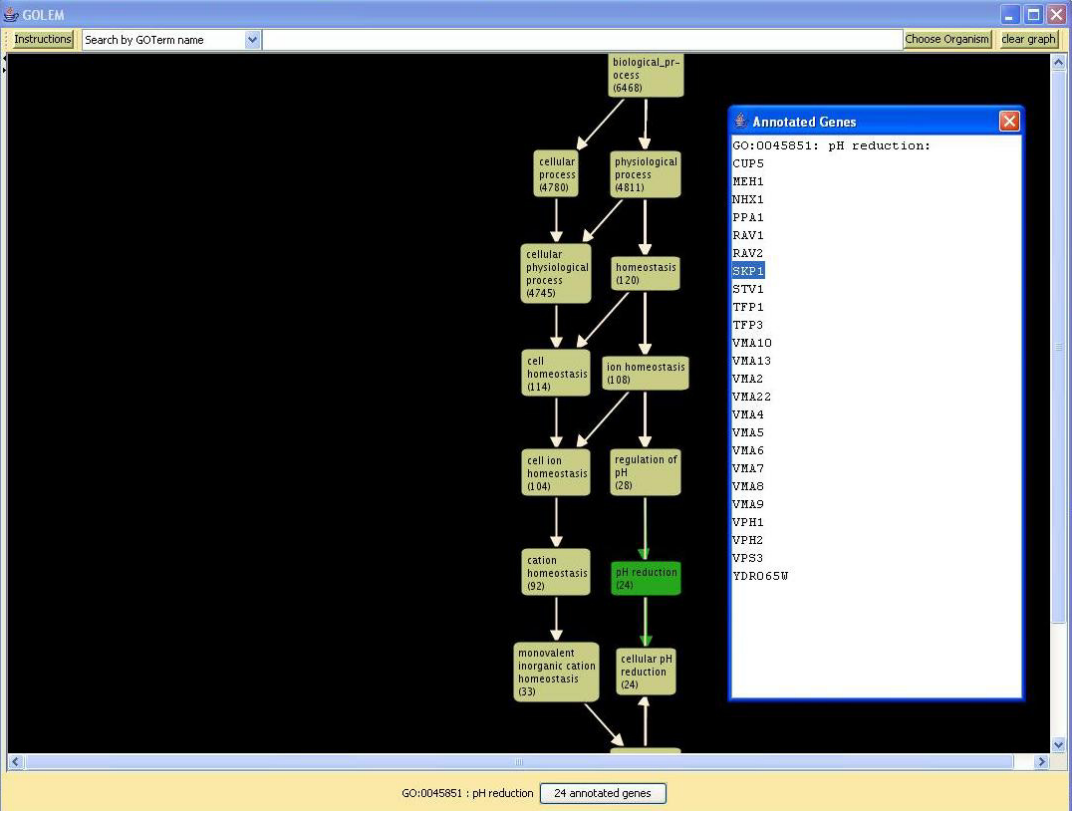
**Displaying Annotated Genes**. When a node is selected, GOLEM displays a button with the number of genes annotated to the node at the bottom of the graph. Clicking on the button displays a list of the genes annotated to this term in the selected annotations file. For example, after selecting the SGD yeast annotations file and the term "pH reduction," the user can view the genes annotated to the GO term "pH reduction" in SGD's yeast annotations file.

Clicking on a node changes the node's color and the color of all adjacent edges, displays the name of the selected node at the bottom of the graph, and shows the number of genes annotated to the node in a button at the bottom of the graph. Clicking on that button provides a complete list of annotated genes (Figure 2). Right-clicking on a node displays a popup menu that allows the user to show the children of the selected node, show the ancestors of the selected node, or focus the graph to show the local structure of the gene ontology hierarchy around that node (Figure 1). GOLEM also permits the user to manually rearrange nodes of the graph in order to improve the appearance of the display.

#### Using local files

The user can choose between using a local gene ontology file defining the GO DAG and downloading the most recent gene ontology file from the Gene Ontology Consortium web page. For setting organism-specific annotations, GOLEM allows the user to choose between selecting from a list of annotations files which are downloaded from the GO web page, and choosing a local annotations file. Choosing a local file typically results in a shorter loading time, while choosing to download the file ensures that GOLEM will use the most recent version of gene ontology data. The applet version of GOLEM reads in the data from a stored cache.

#### Choose annotations

**Figure 3:**
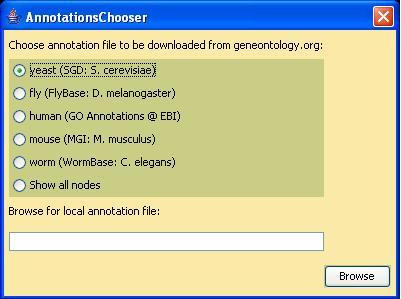
**Choosing Annotations**. GOLEM permits the user either to select a local annotations file or to download an annotations file from the gene ontology web page. After an annotations file is chosen, GOLEM restricts the graph display to terms with annotated genes in the selected annotations file and activates the GeneFinder and Find Enriched Nodes tools.

GOLEM permits the user to select a species-specific annotations file, to browse all nodes, or to select a local annotations file (Figure 3). GOLEM provides built-in access to the following species-specific annotations files: *S. cerevisiae *(SGD), *D. melanogaster *(FlyBase), *M. musculus *(MGI), *C. elegans *(WormBase), and *H. sapiens *(GO Annotations at EBI). All files are read from the gene ontology consortium website. Users who wish to browse the annotations for another species or who prefer to use a local annotations file can input a local file in annotations file format. After an annotations file is selected, GOLEM restricts the graph view to terms that have annotated genes.

### Search capabilities

#### Find enriched nodes

**Figure 4:**
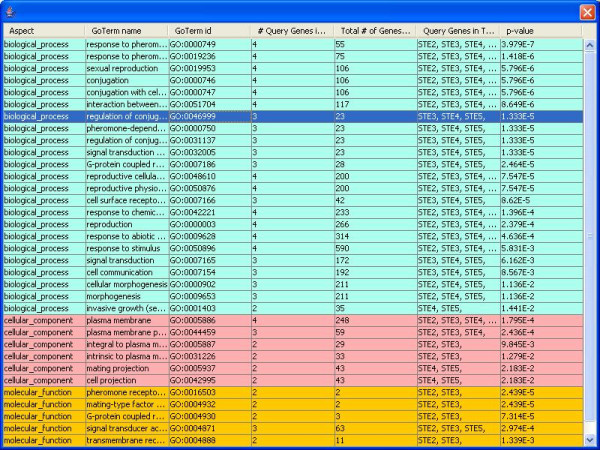
**Finding Enriched Nodes**. The "Find Enriched Nodes" tool displays a table of enriched nodes. Selecting a row of the table displays the DAG structure of the gene ontology hierarchy in the neighborhood of the selected term. All three ontologies (biological process, molecular function, and cellular component) are searched simultaneously, and results are color-coded by ontology and sorted by ontology and p-value. The table shown in this figure resulted from a search for GO terms enriched in the yeast genes STE2, STE3, STE4, and STE5.

GOLEM incorporates a function that finds nodes enriched in a list of input genes (Figure 4). The user enters a list of genes into a text box. The gene list may include either GO DB_Object_Symbols or GO DB_Object_Synonyms as input (in yeast, the DB_Object_Symbol of a gene is typically the gene common name, and the DB_Object_Synonym is the ORF name). GOLEM outputs a table showing the enriched terms. The table displays the name, id, number of annotated query genes, total number of annotated genes, names of annotated query genes, and p-value of each enriched node. Results are sorted by ontology branch (biological process, molecular function, or cellular component, each designated by a different color in the table) and in order of ascending p-values. Selecting a row of the table displays the DAG of the portion of GO containing the selected node in the central display panel window. Selecting multiple rows of the table within the same ontology displays the gene ontology hierarchy centered around all selected terms. The user must first select an annotations file to use this tool (Figure 3).

#### GeneFinder

**Figure 5:**
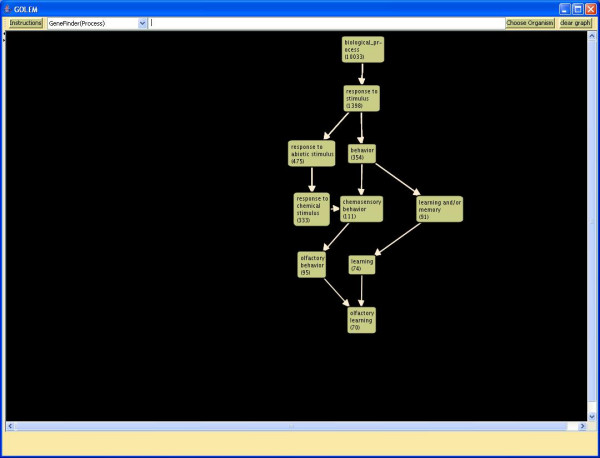
**GeneFinder**. The GeneFinder tool displays all terms in GO to which a selected gene is annotated. Searching for the fly gene ZOL after choosing a fly annotation file displays all terms annotated to this gene.

GOLEM displays all annotated nodes for a given gene within a particular ontology selected by the user (biological process, molecular function, or cellular component). The user must first select an annotations file to use this tool. The GeneFinder tool takes the GO DB_Object_Symbol of a gene as input (in yeast, this is the gene's common name, or ORF if the gene is unnamed). For example, the user could search for all nodes in the biological process ontology annotated to the fly gene ZOL in the FlyBase annotations file. GOLEM would display all nodes to which ZOL is annotated, and the interconnections between these nodes (Figure 5).

#### GOTermSearch

**Figure 6:**
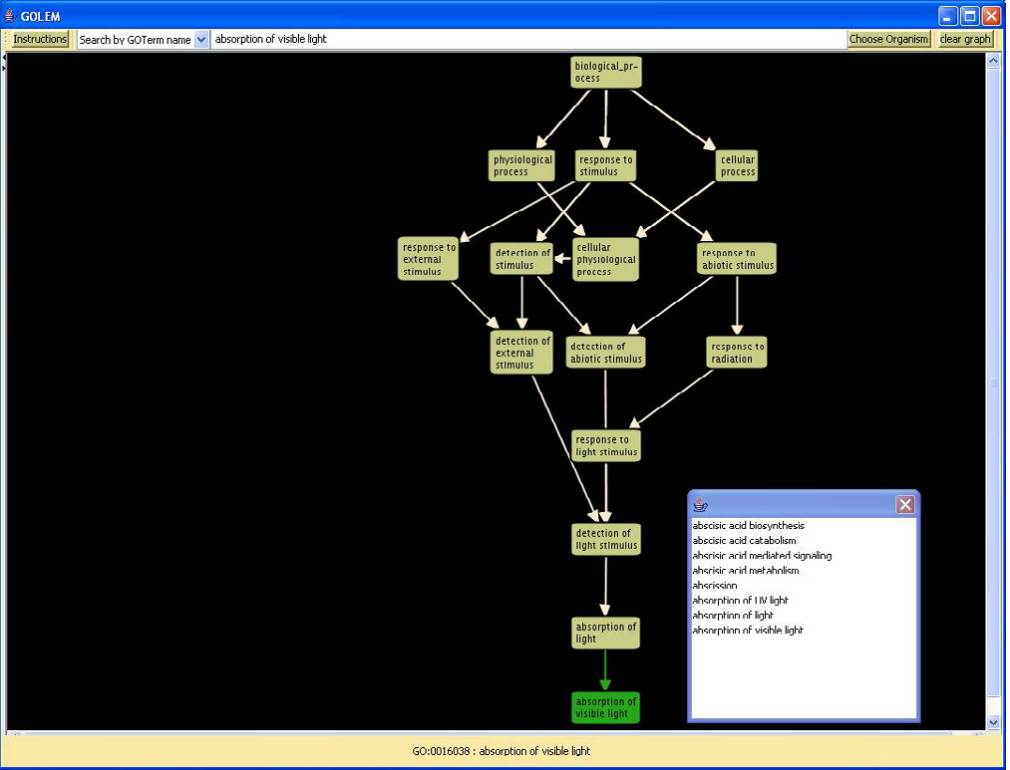
**Search by Go Term Name**. GOLEM's search aid tool allows the user to see all GO terms beginning with the combination of letters that the user has entered. When the user types the letters "abs", the search aid tool lists all GO terms beginning with these letters. Selecting a term in the list displays its name in the text field at the top of GOLEM's display window.

GOLEM is searchable by gene ontology term name. It displays the selected node, one generation of children, and all possible paths back to the root node. As a search aid, a popup window will show all nodes that begin with the combination of letters that the user has entered. Thus, if the user types "oxida", they will see "oxidation of lead sulfide," "oxidative phosphorylation," "oxidative phosphorylation uncoupler activity," and "oxidative photosynthetic carbon pathway" as suggested query choices (Figure 6).

#### GOid search

GOLEM is searchable by the unique gene ontology identification number. It displays the selected node, all possible paths back to the root node, and one generation of child nodes.

### Timing information

The GOLEM application took approximately 5 seconds to initialize the program and load a list of local ontology terms on a 2.5 GHz Celeron CPU with 500 MB of RAM running Microsoft Windows XP. If an update of GO is desired, GOLEM took approximately 40 seconds to initialize and load ontology terms from the geneontology.org web page. Loading of annotations is fast; a local yeast annotation file can be loaded in approximately 2 seconds, for example, and in our trials Internet download of the yeast annotations file took 10 seconds over a broadband connection. After this initial loading time, there was typically none or little perceptible delay when searching the ontology hierarchy by GO name, GO id, or gene name, or when focusing the graph on a specific node, showing the children of a node, showing the ancestors of a node, or finding enriched nodes.

### Sample use case

After loading the yeast annotations file from the gene ontology web page, a biologist interested in the yeast gene SRX1 could use the GeneFinder function to see all of the terms in the biological process ontology to which SRX1 is annotated. One of these terms is "response to oxidative stress." The "Focus Graph" function could then be used to display all children and all paths to the root node from the "response to oxidative stress" node. The researcher could also use the "GOTermSearch" function to find the "response to oxidative stress" term and examine its location in the GO graph as well as the genes annotated to it.

The biologist could then use GOLEM to examine a cluster of genes identified in a microarray study. For example, GOLEM could be used to analyze the MET cluster of genes, which was identified in the yeast cell cycle study by Spellman *et al*. and contains 20 genes [17]. Applying the Bonferroni correction and setting the p-value cutoff at p = 0.05, GOLEM's "Find Enriched Nodes" function returns a table containing 24 enriched biological process nodes, one enriched cellular component node, and 10 enriched molecular function nodes. As GOLEM searches all three ontologies simultaneously, a single search finds all enriched nodes. In this case, the enriched biological process nodes include "sulfur amino acid metabolism" (9 genes), "methionine metabolism" (7 genes) and "sulfur amino acid biosynthesis" (5 genes), supporting Spellman *et al*.'s conclusion that many of the genes in this cluster are involved in methionine metabolism.

A researcher interested in exploring an enriched GO term in more detail can select that term from GOLEM's table of enriched terms, which displays the directed acyclic graph in the neighborhood of the selected term. By selecting multiple terms within the same ontology in the table of enriched nodes, the researcher can view the graph centered around several nodes. This feature allows the user to visualize the relationships in the GO DAG between nodes enriched in the list of input genes. In our example of analyzing the MET cluster, this type of analysis would quickly show both the interrelationships among the enriched terms and relationships to other nodes such as "sulfur metabolism" which is an ancestor of several of the enriched terms. As this example indicates, GOLEM can aid researchers with hypothesis generation and testing, as well as show the broader biological context of those hypotheses.

## Conclusion

GOLEM provides dynamic graph-based visualization of the Gene Ontology hierarchy. It is the only GO visualization tool to provide a dynamically expandable graph display of a local portion of GO. The GOLEM graph view is highly interactive, and can be expanded and focused without network traffic over the server. GOLEM also provides gene set analysis functionality, allowing the user to search for GO terms enriched in genes of interest. Furthermore, the GOLEM application can run without an Internet connection on local ontology and annotations files, although a web-based applet version is also available. In short, GOLEM facilitates rapid, organism-specific searches of the gene ontology and provides a useful tool for biologists to analyze gene sets of interest and to navigate the ontologies' structure.

## Availability and requirements

Project Name: GOLEM

Project Home Page: 

Operating System: Platform-independent

Programming language: Java

Requirements: Java v1.5 or greater

Licence: Open-source

## Authors' contributions

RSGS implemented the software and drafted the manuscript. MAH contributed implementations for some functionality and provided input on the manuscript. CH and CLM provided vital input and insights during the development and testing processes. OGT proposed and supervised the project. All authors read and approved the final manuscript.
